# Post-Attentive Integration and Topographic Map Distribution During Audiovisual Processing in Dyslexia: A P300 Event-Related Component Analysis

**DOI:** 10.21315/mjms2020.27.4.12

**Published:** 2020-08-19

**Authors:** Siti Atiyah Ali, Tahamina Begum, Mohammed Faruque Reza, Nor Asyikin Fadzil, Faiz Mustafar

**Affiliations:** 1 Department of Neurosciences, School of Medical Sciences, Universiti Sains Malaysia, Kubang Kerian, Kota Bharu, Kelantan, Malaysia; 2 Department of Psychiatry, School of Medical Sciences, Universiti Sains Malaysia, Kubang Kerian, Kota Bharu, Kelantan, Malaysia

**Keywords:** event-related potential, P300 component, post-attentive integration, dyslexia, topographic map

## Abstract

**Background:**

Research on audiovisual post-attentive integration has been carried out using a variety of experimental paradigms and experimental groups but not yet studied in dyslexia. We investigated post-attentive integration and topographic voltage distribution in children with dyslexia by analysing the P300 event-related potential (ERP) component.

**Methods:**

We used a 128-child ERP net for the ERP experiment. Two types of stimuli were presented as either congruent or incongruent stimuli. Congruent stimuli included a matching auditory sound with an animal image, whereas incongruent stimuli included unmatched animal sounds. A total of 24 age-matched children were recruited in the control (*n* = 12) and dyslexia (*n* = 12) groups. Children pressed button ‘1’ or ‘2’ when presented with congruent or incongruent stimuli, respectively. The P300 amplitudes and latencies with topographic voltage distribution were analysed for both groups.

**Results:**

The dyslexia group evoked significantly higher P300 amplitudes at the T4 area than the control group. No significant differences were found in cases of P300 latency. Moreover, the dyslexia group demonstrated a higher intensity of P300 voltage distribution in the right parietal and left occipital areas than the control group.

**Conclusion:**

Post-attentive integration for children with dyslexia is higher and that this integration process implicated the parietal and occipital areas.

## Introduction

Audiovisual (AV) integration is fundamental for perception and learning in one’s natural environment (1). The AV system is integrated with the multisensory system which activates in a manner where one sensory system can enhance the other sensory system. This occurs by altering the different sensory inputs whereby attention is then either allocated to the scene or auditory stimuli (2). This cross-modal relationship provides information about the underlying mechanism of human sensory and cognitive control processing.

Research has been conducted using AV integration during reading and by studying the corresponding event-related potential (ERP) data (3, 4). In spelling, reading and writing the alphabet, children develop their multisensory system during the learning process. Learning disabilities including dyslexia develop when this multisensory system is interrupted (5). Children with dyslexia exhibit deficiencies in AV integration (6, 7), which might disrupt attention leading to reading deficiencies (8). According to United Nations Children’s Fund (UNICEF) (9), Malaysia has 165,281 school children with learning disabilities including dyslexia and this number is increasing every year. This has resulted in a greater interest for researchers to study the underlying mechanism of dyslexia on a neuronal level of AV integration, in hopes to improve quality of life for children with dyslexia.

Neuronal recording can be done during single or AV paired stimuli. ERP studies are cheaper, are non-invasive and have high temporal resolutions. Amplitudes and latencies of ERP components provide information about activation of neurons in particular areas of the brain during stimuli presentation (10, 11). P300 is one of the major positive ERP components ranging from 300 ms–800 ms after being evoked by auditory stimulation. Amplitudes and latencies of the P300 component reflect auditory attention, which is cortically distributed at the prefrontal and temporal-parietal areas (12). Central auditory processing was intact in patients with dyslexia, as no significant changes were revealed in P300 amplitudes and latencies (13). Shorter amplitudes and prolonged latencies of P300 were assumed to be a result of the lack of mental workload and delayed processing in dyslexia (14). The outcome of P300 components were all from single auditory stimuli and a variety of experimental paradigms were used among different studies.

Combinations of auditory stimuli with visual stimuli might allow for exploration of attention processing. Pairing AV stimuli together enhances the participant’s ability to respond to such stimuli (15). If AV integration is not synchronised, then a lack of processing of any sensory system might occur in individuals with dyslexia (16). Higher amplitudes of the P300 component were found during congruent (matched AV) stimuli compared to incongruent stimuli (non-matched). These results demonstrated that the P300 component is able to elucidate post-attentive auditory processing during viewing of AV paired stimuli (17). While exploring the functions of sensory systems, topographic mapping can provide important information about brain functions during any stimulus (18). In control participants, the P300 component is typically measured over parietal areas and magnitude depends on the types of cognitive functions (12) and on the stimuli pattern type.

The present study intended to explore the post-attentive integration and topographic distribution of the P300 ERP component during AV stimulus processing in children with dyslexia in Malaysia.

## Methods

We recruited control and children with dyslexia following approval from the Human Ethics Committee of Universiti Sains Malaysia (USM). Sample size was calculated using power and sample size (PS) software with one statistician. True difference in the control and dyslexic means was 1.89 (δ) and standard deviation was 1.56 (σ) (13). Probability power was 0.8 and alpha value was set as 0.05. Therefore, the sample size included twelve in each group (i.e. control participants and children with dyslexia). Control participants were recruited from normal schools and children with dyslexia were recruited from special schools for children with dyslexia located in Kelantan, Malaysia. Permission was obtained from all school authorities and the Ministry of Education in Malaysia. All children in both groups completed the Dyslexic Screening Instrument (DSI) and were assessed by the same clinical psychiatrist. All parents and children provided written inform consent before the sitting experiment in the magnetoencephalography (MEG)/ERP room within the Hospital Universiti Sains Malaysia (HUSM).

### Experimental Set Up

The ERP experiment was done using a children’s 128 ERP net after setting it on the participant’s head. All participants sat in a dimly lit sound treated room and 80 cm away from a 22 inches LCD computer. Stimuli were set up using E-Prime software. All children had normal or corrected to normal vision. Raw data were recorded using Net station software.

**Figure 1:**
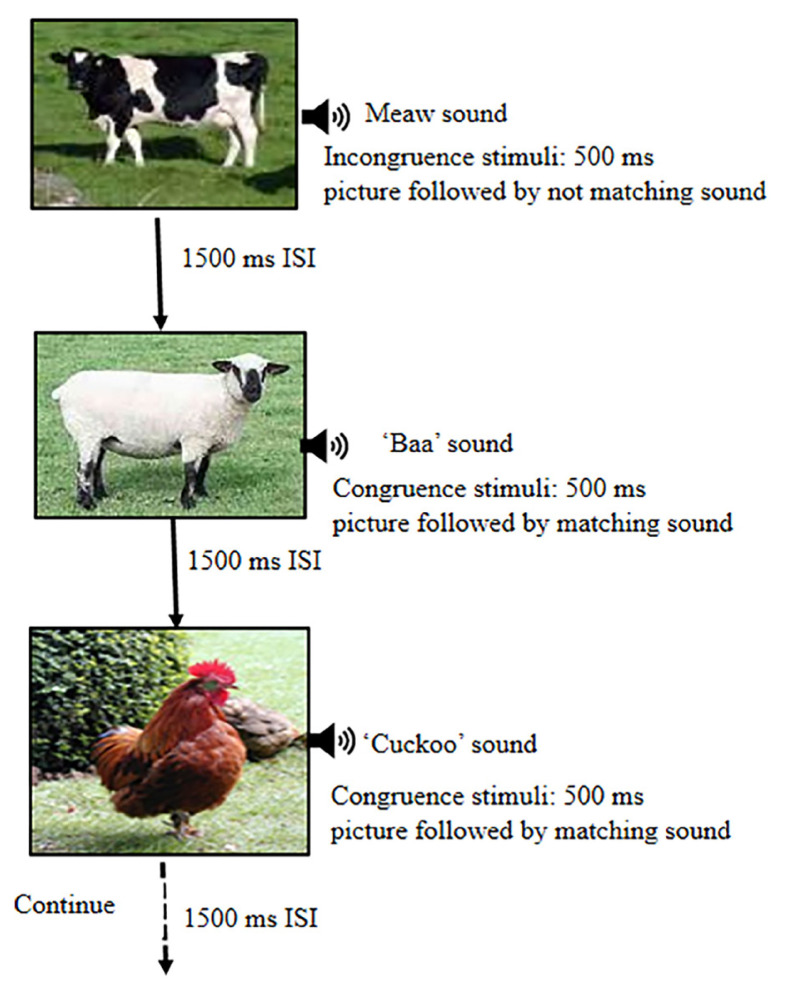
A modified AV, picture-sound matching experimental paradigm was shown where congruent stimuli included matched sound with pictures and incongruent stimuli were unmatched sound with pictures. Each picture and sound pair was presented for 500 ms, and the ISI was 1500 ms

The AV stimuli used included a modified version from a previous study (15). Specifically, images of animals were incorporated as visual stimuli which were matched with the corresponding animal sound (congruent stimuli) by participants. Animal images with un-matched sounds were set up as incongruent stimuli. Children pushed button ‘1’ when viewing congruent stimuli and pushed button ‘2’ when viewing incongruent stimuli. All AV images were 500 ms with an interstimulus interval (ISI) of 1500 ms. There were 100 ms intervals between the presentation of visual images and auditory sounds (Figure 1).

### Statistical Analysis

Statistical analyses were conducted first within the Net station and then SPSS 24 software. Data analysis procedures were mentioned in some previous studies (19, 20). Filtering, segmentation and baseline corrections were done using Net station software which included (0.03–30) Hz, (-100–900) ms, and (-100) ms, respectively. Eye blink, eye movement, and body movement artifacts were removed using artifact detection tools. Amplitudes and latencies of the P300 ERP component were collected during congruence and incongruence stimuli in both groups at 19 electrode sites using the 10–20 system. Later, deducted mean values (incongruence mean value was subtracted from congruence mean value) were collected for comparison. Topographic distribution was conducted using the same software. Statistically significant (i.e. *P* ≤ 0.05) differences were examined using Mann-Whitney U tests in the SPSS 24 software.

## Results

According to demographic information collected from the participants, mean ages in the control (*n* = 12, 7M, 5F) and dyslexia (*n* = 12, 10M, 2F) groups were 10.08 (SD 1.16) years and 10.75 (SD 1.14) years, respectively. There were no significant differences in age between groups. Therefore, participants in both groups were age-matched and were also all right handed.

**Figure 2:**
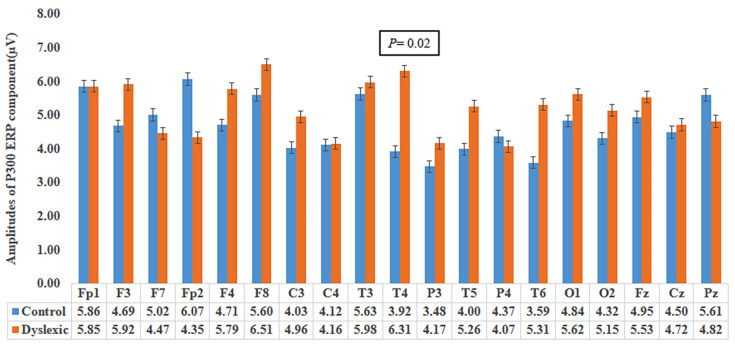
Bar graph showing mean P300 amplitudes between control and dyslexia groups at 19 electrode locations. ‘I’ symbols represent standard error bars.

**Figure 3:**
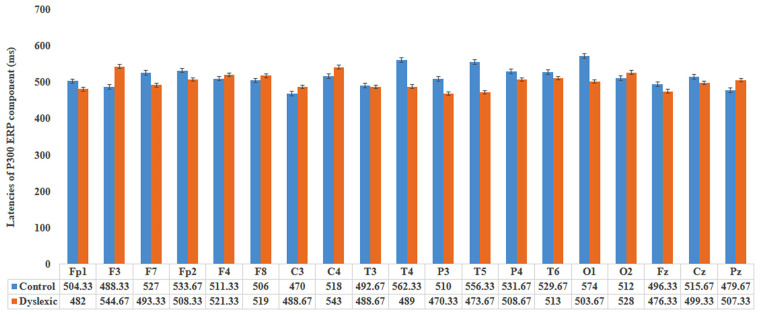
Bar graph showing mean P300 latencies between control and dyslexia groups at 19 electrode locations. ‘I’ symbols represent standard error bars.

Mann-Whitney U tests revealed that the P300 amplitudes were not significant at any electrode locations between groups except at the T4 area. Specifically, the Dyslexia group evoked significantly (*P* = 0.02) higher amplitude (6.31 μV) at the T4 location compared to the control group (3.92 μV). The *P*-values for P300 amplitudes at areas Fp1, F3, F7, Fp2, F4, F8, C3, C4, T3, P3, T5, P4, T6, O1, O2, Fz, Cz and Pz were: 0.85, 0.22, 0.71, 0.06, 0.18, 0.38, 0.30, 0.93, 0.63, 0.30, 0.55, 0.77, 0.09, 0.52, 0.74, 0.38, 0.99, 0.46, respectively (Figure 2). There were no significant differences of P300 latencies at any channels between groups. The *P*-values were: 0.58, 0.21, 0.54, 0.57, 0.87, 0.81, 0.60, 0.67, 0.93, 0.11, 0.47, 0.06, 0.58, 0.78, 0.07, 0.74, 0.65, 0.79, 0.43 at Fp1, F3, F7, Fp2, F4, F8, C3, C4, T3, T4, P3, T5, P4, T6, O1, O2, Fz, Cz and Pz locations, respectively (Figure 3).

**Figure 4:**
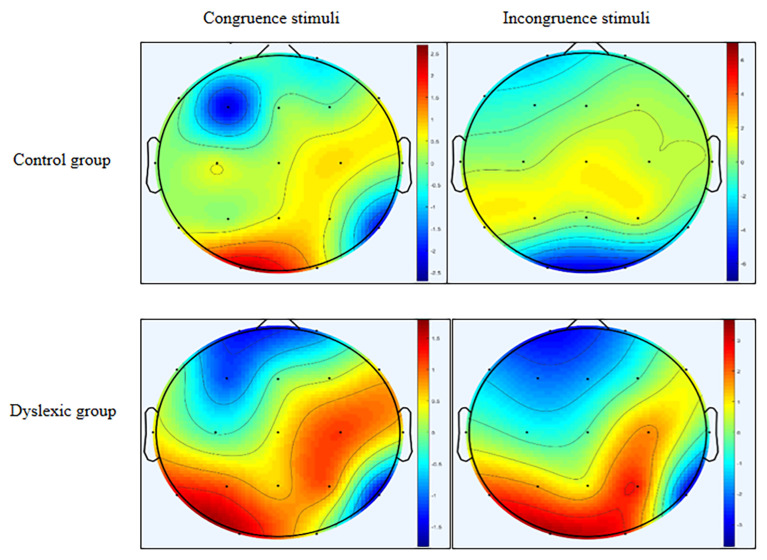
A topographic distribution of the P300 ERP component at 383 ms during congruent and incongruent stimuli in control and dyslexia groups. Red areas within the colour bar indicated positive voltage activity of the P300 component

Topographic scalp distribution revealed that P300 distributions were in the right parietal and left occipital areas during viewing of both congruent and incongruent stimuli in both the control and dyslexia groups. The only difference was the prominent deeper intensity (where the red colour reflects P300 voltage activity) in the dyslexia group for both stimuli types, in comparison to the control group (Figure 4).

## Discussion

In this study, post-attentive integration was assessed during AV stimuli viewing in children with dyslexia by analysing amplitudes and latencies of the P300 ERP component. Topographic distributions of the P300 component were also evaluated. Results revealed that children with dyslexia had significantly higher amplitudes at the T4 area compared to the control group. Additionally, children with dyslexia had a higher intensity of P300 voltage distribution in the right parietal and left occipital areas of the brain.

Higher P300 amplitude has been observed during improvement of auditory information processing when AV stimuli are paired among musicians. In this case, the visual context helped enhance auditory sensory processing due to post attention integration (17). Visual contexts influence the auditory sensory system by taking attention away during reading (21). Higher auditory P300 amplitudes during viewing of AV stimuli reflected cross-modal attention during auditory sensory processing (22). In our study, we asked children to pay attention toward the auditory sound and identify the matched (i.e. congruent stimulus) or unmatched animal sound (i.e. incongruent stimulus) with a previously shown image of an animal. Children with dyslexia showed significantly higher P300 ERP amplitudes in the T4 area in comparison to the control group. The previously interpreted results (Figure 2) (17, 21, 22) revealed that our dyslexia group had good post-attentive integration while experiencing AV stimuli where visual animal images enhanced the auditory sensory system (17) and provided more focus toward the auditory sound to match it with the image (21, 22). Our result of higher amplitude of P300 component at T4 area in dyslexia group might be due to enhanced phonological awareness for using extra listening device during study time in their special school which leads to auditory neuroplasticity (23).

The P300 component, which reflects good post-attention integration during AV processing, can give us more information about the auditory P300 topographic distribution in children with dyslexia. Children with dyslexia exhibit higher P300 voltage activities in the left occipital and right parietal areas during processing of both congruent and incongruent stimuli in comparison to control children (Figure 4). Higher P300 voltage activities suggested the possibility of higher attention (24) being manifested during the AV processing, regardless of the stimulus congruency type. This may be evidence suggesting a compensatory mechanism present within cognitive deficits. The parietal lobe has an important role for integrating sensory information between different sensory systems (25). The occipital lobe can process perception of complex image stimuli (26). Taken together, this information drives us to the conclusion that during AV stimuli processing (irrespective of congruency), the occipital lobe processes the perception of animal images as a visual sensory input (26). This in turn enhances post-attentive integration (24) during hearing of auditory stimuli in the parietal lobe (25) in children with dyslexia. However, source localisation of the post-attentive P300 component can provide more information for a future study.

## Conclusion

We investigated post-attentive integration during AV processing by analysing the P300 ERP component in children with dyslexia using topographic P300 voltage distribution. We concluded that children with dyslexia have good post-attentive integration during AV processing (irrespective of whether the stimuli were congruent or not) due to sensory integration between occipital-parietal areas.
